# Effect of Biosynthesized Silver Nanoparticles on Bacterial Biofilm Changes in *S. aureus* and *E. coli*

**DOI:** 10.3390/nano12132183

**Published:** 2022-06-25

**Authors:** Bozena Hosnedlova, Daniil Kabanov, Marta Kepinska, Vedha Hari B Narayanan, Arli Aditya Parikesit, Carlos Fernandez, Geir Bjørklund, Hoai Viet Nguyen, Awais Farid, Jiri Sochor, Agnes Pholosi, Mojmir Baron, Milan Jakubek, Rene Kizek

**Affiliations:** 1 BIOCEV, First Faculty of Medicine, Charles University, Průmyslová 595, CZ-25250 Vestec, Czech Republic; bozena.hosnedlova@post.cz (B.H.); milan.jakubek@lf1.cuni.cz (M.J.); 2 Department of Paediatrics and Inherited Metabolic Disorders, First Faculty of Medicine, Charles University and General University Hospital in Prague, Ke Karlovu 455/2, CZ-128 08 Prague, Czech Republic; 3 Department of Viticulture and Enology, Faculty of Horticulture, Mendel University in Brno, Valticka 337, CZ-691 44 Lednice, Czech Republic; dac100@mail.ru (D.K.); jiri.sochor@mendelu.cz (J.S.); mojmir.baron@mendelu.cz (M.B.); 4 Department of Pharmaceutical Biochemistry, Division of Biomedical and Environmental Analyses, Faculty of Pharmacy, Wroclaw Medical University, Borowska 211a, 50-556 Wroclaw, Poland; marta.kepinska@umed.wroc.pl; 5 Department of Pharmacy, School of Chemical and Biotechnology, SASTRA Deemed University, Thanjavur 613-401, India; vedhahari@scbt.sastra.edu; 6 Department of Bioinformatics, School of Life Sciences, Indonesia International Institute for Life Sciences, JI. Pulomas Barat Kav. 88, Jakarta Timur 13210, Indonesia; arli.parikesit@i3l.ac.id; 7 School of Pharmacy and Life Sciences, Robert Gordon University, Garthdee Road, Aberdeen AB10 7QB, UK; c.fernandez@rgu.ac.uk; 8 Council for Nutritional and Environmental Medicine (CONEM), Toften 24, 8610 Mo i Rana, Norway; bjorklund@conem.org; 9 Research Center for Environmental Monitoring and Modeling, University of Science, Vietnam National University, 334 Nguyen Trai Street, Hanoi 100000, Vietnam; nguyenviethoai@hus.edu.vn; 10 Division of Environment and Sustainability, Hong Kong University of Science and Technology, Room 4412, Clear Water Bay, Kowloon, Hong Kong, China; awais@ust.hk; 11 Biosorption and Wastewater Treatment Research Laboratory, Department of Chemistry, Faculty of Applied and Computer Sciences, Vaal University of Technology, P. Bag X021, Vanderbijlpark 1900, South Africa; agnesp@vut.ac.za

**Keywords:** antimicrobial activity, nanoparticles, tropical plant, *L. indica*, *A. scholaris*, *A. multifolium*, plant extracts, *S. aureus*, *E. coli*

## Abstract

One approach for solving the problem of antibiotic resistance and bacterial persistence in biofilms is treatment with metals, including silver in the form of silver nanoparticles (AgNPs). Green synthesis is an environmentally friendly method to synthesize nanoparticles with a broad spectrum of unique properties that depend on the plant extracts used. AgNPs with antibacterial and antibiofilm effects were obtained using green synthesis from plant extracts of *Lagerstroemia indica* (AgNPs_LI), *Alstonia scholaris* (AgNPs_AS), and *Aglaonema multifolium* (AgNPs_AM). Nanoparticles were characterized by transmission electron microscopy (TEM) and energy-dispersive X-ray spectroscopy (EDX) analysis. The ability to quench free radicals and total phenolic content in solution were also evaluated. The antibacterial activity of AgNPs was studied by growth curves as well as using a diffusion test on agar medium plates to determine minimal inhibitory concentrations (MICs). The effect of AgNPs on bacterial biofilms was evaluated by crystal violet (CV) staining. Average minimum inhibitory concentrations of AgNPs_LI, AgNPs_AS, AgNPs_AM were 15 ± 5, 20 + 5, 20 + 5 μg/mL and 20 ± 5, 15 + 5, 15 + 5 μg/mL against Gram-positive (*Staphylococcus aureus*) and Gram-negative (*Escherichia coli*) bacteria, respectively. The *E. coli* strain formed biofilms in the presence of AgNPs, a less dense biofilm than the *S. aureus* strain. The highest inhibitory and destructive effect on biofilms was exhibited by AgNPs prepared using an extract from *L. indica*.

## 1. Introduction

**Figure 1:**
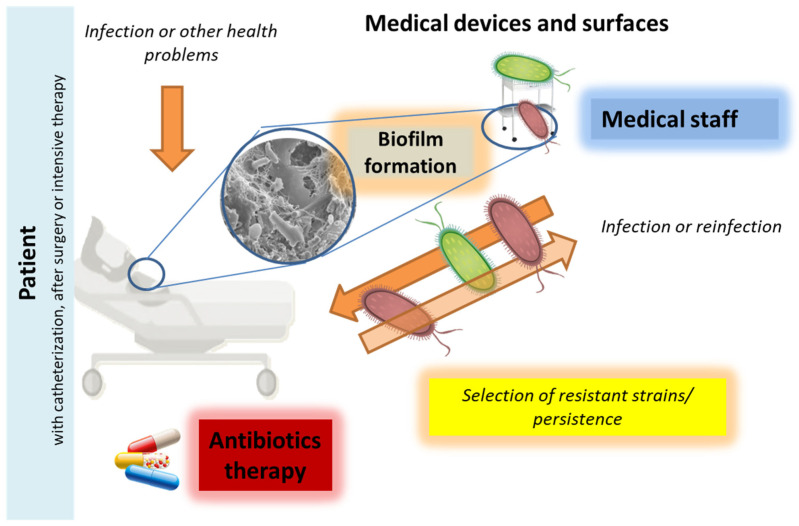
The simplified cycle of nosocomial bacterial infection and the crucial role of biofilms in the spreading and resistance process. The biofilms formed may be related to bacterial resistance depending on environmental conditions, in particular cleaning procedures, disinfection, and antibacterial agents. Increased persistence and avoidance of treatment with antibacterial substances lead to the preservation of bacteria on medical devices, surfaces, and tissues. This increases the risk of reinfection, contributes to the selection of resistant strains and their spread in the hospitals.

Bacterial infections with antibiotic resistance (mainly nosocomial) (Figure 1) continue to be a major problem in the early 21st century [1,2,3] due to the misuse of antibiotics, disinfection in hospitals, and poorly understood mechanisms to avoid treatment [4]. The rapid increase in bacterial resistance requires the search for new strategies. The use of nanomaterials could be one of them. Recently, combinations of antimicrobial polymers with nanomaterials and bioactive substances have also been tested to improve biocidal therapy [5].

Silver nanoparticles (AgNPs) exhibit antimicrobial, antiviral, and antifungal effects [6,7,8]. Mohanta et al. demonstrated strong antibacterial and antibiofilm activity of phyto-synthesized AgNPs against different clinically important human pathogens [5]. Due to their antibacterial properties, AgNPs possess a range of biomedical applications. For example, creams and ointments can incorporate AgNPs for wound healing applications. Silver-doped self-assembling di-phenylalanine hydrogels have been demonstrated to be clinically effective as wound dressing biomaterials. Bandages, gauzes, sutures, and plasters can be functionalized with AgNPs. The efficacy of silver nanocoatings applied to textile materials for wound dressing in preventing bacterial adhesion and biofilm formation has been demonstrated. AgNPs incorporated into wound dressing fabrics showed significant antibacterial effect against both Gram-positive and Gram-negative bacteria and inhibited biofilm formation [9,10,11,12,13,14]. Biofilm is a complex of surface-bound microbial cells that are surrounded by a matrix of extracellular polymers [15].

The biofilm formation by microorganisms (Figure 1) is a mechanism for colonization, virulence, and resistance in the form of cell communities on various surfaces [5]. Biofilm is characterized by a three-dimensional structure (microcolonies or mature biofilm fields) where the cells are placed in the extracellular matrix, which consists mainly of polysaccharides, proteins, and DNA [16]. It has been estimated that biofilm cells can be up to 10,000 times more resistant to antibiotics than planktonic cells [17]. Dispersion as the final step of the biofilm life cycle is considered a promising way to control biofilm [18].

**Figure 2:**
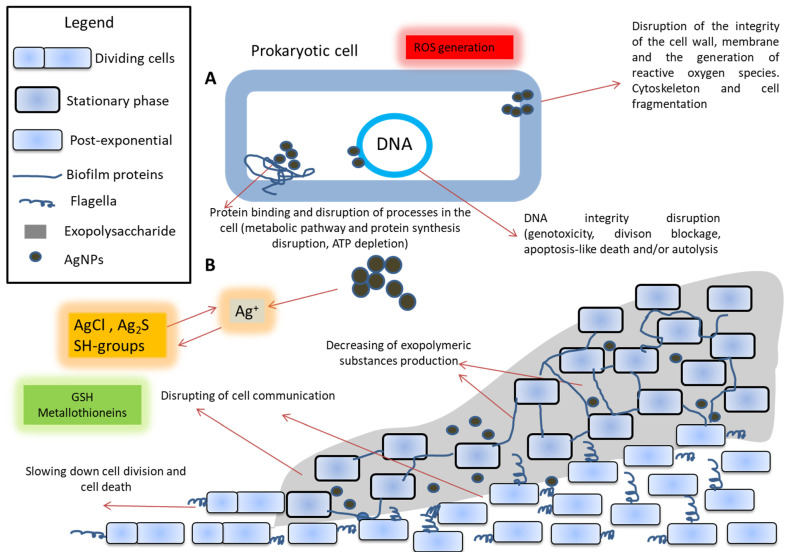
The presumed effect of silver nanoparticles (AgNPs) on prokaryotic cells (**A**) and biofilms (**B**). Silver ions can be released from silver nanoparticles in the environment. These ions can react with chlorides to form AgCl, with sulfur to form Ag_2_S, with the SH groups of glutathione or metallothionein. Silver nanoparticles act on the cell, disrupting the processes of regulation, protein synthesis, ATP depletion, and damaging the cell wall. Additionally, an increased level of free radicals and a disruption of the integrity of DNA, division, blockage, apoptosis-like death and/or autolysis are observed. All this results in a violation of the structure and the synthesis of the components of biofilms. Additionally, it leads to disrupt intercellular signaling and direct cell death in the biofilm [26,27].

The effects of nanoparticles on biofilms can be divided into two categories: (a) prevention of biofilm formation (when nanoparticles are present in media during cultivation) and (b) biofilm destruction [19,20]. Silver has a dose-dependent effect on bacterial cells and biofilms, but its efficiency is lower for biofilms than for planktonic (non-adhered) cultures. However, sublethal doses of AgNPs can increase the production of both biofilm matrix polysaccharides and proteins compared to the control, which significantly changes the biofilm structure [21]. On biofilms, nanoparticles decrease bacterial metabolic activity due to the disruption of intracellular processes. Changes due to this occur in the formation of microcolonies and the maturation of biofilms [22]. Particle size, synthesis method, and exposure time are crucial for the effect of nanoparticles on biofilms [23,24]. Additionally, nanoparticles can accumulate in a biofilm and change its physical properties (mechanical stability), including an impact on the total density [25]. The general scheme of the action of nanoparticles on biofilms is presented in Figure 2.

Antibacterial action of AgNPs is mediated by the following mechanisms: direct contact with the bacteria components, release of bioactive ions, disruption of some metabolic pathways, formation of reactive oxygen species (ROS), genotoxicity, inhibition of bacterial DNA replication, alteration in the wall and cytoplasm of the cell, alteration in membrane permeability of bacteria and ionic change [28,29,30,31,32,33,34,35]. These mechanisms are primarily mediated by the action of the AgNPs. The main antibacterial effect is based on the release of Ag^+^ ions from AgNPs [36]. The antibacterial mechanisms of AgNPs utilize targeting many bacterial cell components [37] such as bacterial wall (disruption and/or the membrane permeability increase), tRNA, the inactivation the respiratory chain (ATP depletion), enzyme and protein synthesis as well as DNA-binding (inhibition of replication) [32,33,34]. The overall bactericidal efficacy of AgNPs depends not only on the rate of Ag^+^ ions generation but also on the size, shape and total surface area of AgNPs and type of coating/corona [28].

It is supposed that the difference in the efficiency of AgNPs against different types of bacteria (Gram-positive, Gram-negative, acid-fast bacteria) primarily depends on the structure and thickness of bacterial cell walls [38,39,40,41].

Silver nanoparticles generate free radicals, disrupt the integrity of the cell membrane and bind to proteins and DNA. The growth of the cell population can slow down, and cells can die from the action of silver [23]. Meanwhile, interest in studying the action of AgNPs on bacteria under various conditions is due to a growing number of reports of an increase in resistance to heavy metals and various antibacterial substances based on metal ions or nanoparticles. This also applies to selective mechanisms, various cellular and pleiotropic effects in the population associated with the action of metals [42]. In this regard, it is also important to look for new approaches to the development of nanoparticles with new physical and antibacterial properties as potential antibiotic agents. For example, different ways of how to prepare various AgNPs-based nanotransporters for targeted therapy of tumors or bacterial diseases are being sought. The antibacterial properties of AgNPs are the most studied and discussed [43,44]. However, their biological effect is still not very clear and must be carefully studied [45,46]. Indeed, AgNPs are characterized not only by antibacterial but also antiviral, antifungal [47], and antitumor effects [48].

In addition to bacteria and animals, green synthesis also uses plants. Plant extracts replace chemical reducing agents in the reaction, such as NaBH_4_ or LiAlH_4_. Many tropical and subtropical plant species contain a lot of biologically important molecules (including peptides, proteins, and nucleic acids). Phenolic compounds are especially important for antibacterial activity. Crape myrtle (*Lagerstroemia indica*) is an ornamental woody plant used in medicine. Its extract contains many different alkaloids and flavonoids and has anti-inflammatory and antimicrobial effects [49,50,51,52]. Blackboard tree *(Alstonia scholaris)* has been used in traditional medicine primarily for the treatment of respiratory and inflammatory diseases, malaria, jaundice, and gastrointestinal disorders. Evidence-based medicine has confirmed the protective effect of its extract on respiratory tract tissue during inflammation; four main alkaloids (scholaricine, 19-epischolaricine, vallesamine, picrinine) from the extracts reduced post-infection cough in mice [53,54,55]. Pentacyclic triterpenoids from extracts of *A. scholaris* have antitumor [50] and synergistic antibacterial [56], antiallergic [55,57] and antihypertensive effects [58]. *Aglaonema multifolium* is an evergreen ornamental plant, common in the Asian region. In some species, e.g., *Aglaonema* spp., however, the content of compounds that have significant toxic effects on eukaryotic cells has been demonstrated [59,60].

This work aims to present a green synthesis method for AgNPs with a modified surface to increase their antibacterial ability while evaluating their effect on biofilms and testing their activity. The use of plant extracts replaces chemical reducing agents in the reaction, such as NaBH_4_. In addition, tropical and subtropical plants contain a lot of biologically important molecules, especially phenolic substances, important in antibacterial activity.

## 2. Materials and Methods

### 2.1. Chemicals

Silver nitrate, methanol, NaCl, and other chemicals (KCl, NaCl, AgNO_3_, boritan, NaHPO_4_, NaH_2_PO_4_, etc.) used were purchased from Merck (Darmstadt, Germany) with a purity of 99%. For LB media preparation, 10 g/L tryptone, 5 g/L yeast extract (Duchefa Biochemie B.V., Haarlem, The Netherlands), and 10 g/L NaCl (Merck, Darmstadt, Germany) were used.

### 2.2. Instruments

#### 2.2.1. Spectrophotometric Analysis

Spectrophotometry: a UV-Vis UV-3100PC, VWR (Radnor, PA, USA) single-beam spectrophotometer was used to record the UV-Vis spectra. The Vis spectrum was measured every 2 nm in the range of 400–800 nm in plastic cuvettes with an optical path of 1 cm. An Infinite F50 (Tecan, Männedorf, Switzerland) was used for measurement on a polystyrene microtiter plate (Gama Group a.s., Ceske Budejovice, Czech Republic). Automated spectrometric measurements: BS-300 chemical analyzer from Mindray (Shenzhen, China), cuvettes 5 mm × 6 mm × 30 mm, optical path 5 mm, and a volume of the reaction mixture in the cuvette 180–500 µL were used. Reagents and samples were placed on the cooled sample holder (4 °C) and automatically pipetted directly into plastic cuvettes. Incubation proceeded at 37 °C. The mixture was consequently stirred. The washing steps by ultrapure water (18 mΩ) were carried out in the middle of the pipetting.

#### 2.2.2. Other Devices

Deionized water was prepared using reverse osmosis equipment Aqual 25 (AQUAL s.r.o., Brno, Czech Republic) and subsequently treated to an 18 MΩ purity by an ELGA deionizer from Purlab Flex (London, UK). Conductivity and pH were measured with an MU 6100L multimeter from VWR (Radnor, PA, USA). The pH-electrode (662-1161 Phenomenex pH electrode pH 0–14/3M KCl, VWR, Torrance, CA, USA) was regularly calibrated with two-point calibration (VWR buffers, at 22 °C).

### 2.3. Preparation of Silver Nanoparticles by Green Synthesis

Biological material collection sites have been selected in cooperation with CeMM (The Center for Molecular Medicine) researchers. Plant samples were collected in the university campus of the VNU University of Science, Thong Nhat and Tuoi Tre part, Hoan Kiem Lake, and Hanoi Botanical Garden, Hanoi Night Market, Tu Dung Homestay Botanic Garden in Can Tho and Cat Tien National Park in Vietnam. All activities were handled in direct collaboration with workplaces at Vietnamese universities. Plants used in experiments were chosen as follows: *L. indica*, *A. scholaris*, and *A. multifolium*. Individual plant species were classified into families and genera primarily by the appearance of their leaves, stems, and overall plant appearance. Alternatively, flowers or fruits were used to identify plants. Plant identification was provided by the Department of Pharmacognosy of the Faculty of Pharmacology at Tra Vinh University. Plants were dried at 60 °C for 48 h, homogenized by grinding to a particle size of 1–2 mm. Preparation of extract: the mixture was stirred in water (80 °C) for 60 min in a ratio (5 DW g/100 mL, *v/w*). Then, extracts were filtered. Subsequently, centrifugation (15 min, 4000× *g*) was performed. The leachate was mixed with 0.1 M AgNO_3_ (1:1). Silver nitrate and extracts were stirred at room temperature for 24 h at 150 rpm. The formed particles were purified with methanol (1:1). After precipitation, samples were centrifuged and the methanol was pipetted away; synthesized AgNPs were dried (24 h, 60 °C, VWR dryer, Radnor, PA, USA).

### 2.4. HRTEM and Energy-Dispersive X-ray Spectroscopy (EDX)

The nanostructure and surface morphology of the prepared AgNPs was characterized by high-resolution transmission electron microscopy (HRTEM) conducted on a JEOL 2100 HRTEM instrument (JEOL, Tokyo, Japan). The nanomaterials were mixed with absolute ethanol (1:1) in vials and sonicated for 10 min. Carbon grids of 10 μm mesh size were then immersed in the solution containing the nanomaterials, dried, and applied for the analysis. The determination of the individual elemental components of AgNPs was performed using the energy-dispersive X-ray spectroscopy (EDX).

### 2.5. Zetasizer Analysis of Silver Nanoparticles

The size distribution (i.e., the hydrodynamic diameter, DH) was determined by dynamic light scattering (DLS) using the Zetasizer Nano ZS ZEN3600 (Malvern Instruments, Malvern, Worcestershire, UK) with a detection angle of 173° in optically homogeneous square polystyrene cells (Malvern, catalogue no. DTS0012). The samples were diluted a hundredfold with deionized water. All measurements were performed at 25 °C. Each value was obtained as an average of 3 runs with at least 10 measurements. The particle charge (ζ-potential) was measured by the microelectrophoretic method using the same device as the size distribution. All the measurements were performed at 25 °C in polycarbonate cuvettes (Malvern, catalogue no. DTS1070). Each value was obtained as an average of 3 subsequent runs of the instrument with at least 20 measurements.

### 2.6. Electrochemical Measurement

Electrochemical measurements were performed with an AUTOLAB Analyser (Metrohm, Herisau, Switzerland) connected to VA-Stand 663 (Metrohm, Herisau, Switzerland), using a standard cell with three electrodes. The working electrode was a hanging mercury drop electrode (HMDE) with a drop area of 0.4 mm^2^. The reference electrode was an Ag/AgCl/3 M KCl electrode, and the auxiliary electrode was a graphite electrode. The supporting electrolyte was prepared by mixing buffer components. The samples analyzed by differential pulse voltammetry (DPV) were deoxygenated prior to measurements by purging with argon (99.999%) saturated with water for 20 s. In our studies, the Brdicka supporting electrolyte contained 1 mM Co(NH_3_)_6_Cl_3_, 1 M NH_3_(aq), and 1 M NH_4_Cl, pH = 9.6; a surface-active agent was not added. The DPV Brdicka reaction parameters were as follows: an initial potential of –0.6 V, an end potential –1.85 V, a modulation time 0.057 s, an interval of 0.2 s, a step potential of 1.05 mV/s, modulation amplitude of 250 mV, and accumulation time of 240 s [61].

### 2.7. Measurement of Antioxidant Activity of Silver Nanoparticles

Characterization of surface modifications of nanoparticles was performed by methods that were previously optimized [14,52,62,63,64,65,66]. The determination of the antioxidant activity of nanoparticles was performed by methods previously optimized using a BS-300 Chemical analyzer from Mindray (Shenzhen, China). Ferric-reducing antioxidant power (FRAP) is based on the reduction of 2,4,6-tripyridyl-s-triazine (TPTZ) with FeCl_3_·6H_2_O. The reagent was prepared from several solutions: solution 1 was prepared from 1:10 mmol/L solution of TPTZ was dissolved in 40 mmol/L of hydrochloric acid. Solution 2 of 2:20 mmol/L of ferric chloride hexahydrate was prepared in ACS water. Solutions 3 was 3:20 mmol/L acetate buffer, pH 3.6. These three solutions (TPTZ, FeCl_3_, acetate buffer) were mixed in a 1:1:10 ratio. A 200 μL volume of reagent was injected into a plastic cuvette with the subsequent addition of a 4 μL sample. Absorbance was measured at 605 nm for 12 min. The radical of 2,2′-azino-bis(3-ethylbenzothiazoline-6-sulfonic acid)—(ABTS, 7 mM) and potassium peroxodisulfate (5 mM) were mixed in water. The solutions were then prepared by diluting with water in a ratio of 1:9 *v*/*v*, stored for 12 h in the dark at 4 °C before use. A 200 µL volume of reagent (7 mM ABTS^•^ 2,2′-azino-bis(3-ethylbenzothiazoline-6-sulfonic acid (and 4.95 mM potassium peroxodisulfate) were mixed with 4 µL of the sample. Absorbance was measured at 660 nm for 12 min. The 2,2-diphenyl-1-picrylhydrazyl (DPPH) method is based on quenching the color of the radical. A 200 µL volume of reagent (0.095 mM 2,2-diphenyl-1-picrylhydrazyl—DPPH^•^) was incubated with 20 µL of the sample. Absorbance was measured at 505 nm for 12 min. The output ratio of the three mentioned antioxidant methods was achieved by a difference in absorbance at the last (12th) minute and second minute of the assay procedure. Calibration curves were prepared using different concentrations of gallic acid [65].

#### 2.7.1. Total Phenolic Content Determination

Total phenolic content in AgNPs was determined according to Zoufan et al. [66], with minor modifications using a BS-300 Chemical analyzer from Mindray (Shenzhen, China). First, 316 µL of R1 reagent (3.75% Folin-Ciocalteau reagent in ultrapure water) was pipetted into the plastic cuvettes, followed by 4 µL of the measured sample. The reaction was started by adding 80 µL of R2 reagent (10% Na_2_CO_3_ in ultrapure water). Absorbance was measured for 12 min at 670 nm. The output ratio was achieved by a difference in absorbance at the last (12th) minute and second minute of the assay procedure. A calibration curve was prepared using different concentrations of gallic acid. Total phenolic content was expressed as µg of gallic acid equivalent/mL (µg GAE/mL) [67].

#### 2.7.2. Total Protein Amount

Total protein was determined by the biuret method using a BS-300 Chemical analyzer from Mindray (Shenzhen, China). The biuret method is a test used for detecting the presence of peptide bonds. In the presence of peptides, a copper (II) ion forms a violet-colored complex in an alkaline solution. A 200 µL volume of biuret reagent (100 mM potassium sodium tartrate, 100 mM sodium hydroxide, 15 mM potassium iodide and 6 mM copper sulfate) was pipetted into a plastic cuvette with the subsequent addition of 4 µL of the sample. Absorbance was measured at λ = 546 nm after 10 min of incubation. The resulting value was calculated from the absorbance value of the pure biuret reagent and from the absorbance value after 10 min of incubation with the sample.

### 2.8. Antibacterial Activity of Silver Nanoparticles

*Escherichia coli* and *Staphylococcus aureus* were obtained from the Collection of Microorganisms of Masaryk University (Brno, Czech Republic). Cultivation of microorganisms was carried out in LB medium at 37 °C; for experiments overnight (18–24 h) culture was used. Growth curves were determined for 24 h at 25 °C in a sterile microtiter plate (0.25 mL, shaking 5 s, 150 rpm). The measurement was performed at Infinite 50 (Tecan, Männedorf, Switzerland) at 620 nm. Absorbances were recorded every 15 min. The curve area was determined for each concentration in the tested range (5, 10, 15, 20, 25, and 30 μg/mL); the effect was calculated relative to the negative control (well without antibacterial substance). A standard diffusion test was performed on an agarized LB medium based on EUCAST rules with slight modifications [68]. Ten microliters of a solution of nanoparticles with a concentration of 5, 2.5, 1.25, 6.25 mg/mL were placed on the surface of a Petri dish with agar (3%) and incubated at 25 °C. Incubation was carried out for 18 h, plates were photographed, and the area of inhibition zones were measured using the Qinslab color-test. As a control, sterile ultrapure water was used.

### 2.9. Experiments with Biofilms

#### 2.9.1. Biofilm Growth in Liquid Medium and Incubation with Silver Nanoparticles

Biofilms were obtained using a polystyrene sterile 96-well plate. A sterile LB medium and overnight culture of bacterial cells in a final concentration *OD*_600_ = 8 × 10^−5^ were added into the well in a volume of 0.25 mL. During optimization steps of biofilm growth, classic biofilm protocol [69] was modified to achiEve 0.5 McFarland cell concentration diluted close to EUCAST protocol for MIC detection [70].

In control samples without the presence of bacteria and without the presence of nanoparticles in bacteria, various nanoparticles were added to the well before inoculation of the bacterial suspension. The range of tested concentrations was obtained by double dilutions of the nanoparticles in the medium. As a negative control, biofilm without nanoparticles was used. After incubation for 48 h to test the ability of AgNPs to destroy biofilms, mature biofilms were poured with a solution of nanoparticles in Milli-Q water. Biofilms were incubated at 37 °C. As a negative control, a well with Milli-Q water was used. For data processing, values of negative controls were also used for a well with nanoparticle solution and a well with a solution of nanoparticles, bacteria, and 96% ethanol. As an additional negative control, a well with nanoparticles and 96% ethanol was used. To take into account the negative control, the control value was subtracted from the absolute values; the results were presented as a positive value in percentage. A biofilm without nanoparticles was taken for 100%.

#### 2.9.2. Crystal Violet Assay

Crystal violet (CV, Merck, Darmstadt, Germany) staining was used according to O’Toole [69] with some changes to determine the total density of the biofilm. The biofilms on the plate were washed with a stream of 200 mL of sterile tap water and dried for 5 min. Then, 200 μL of crystal violet at a concentration of 0.5% was added to the well. Staining was carried out at room temperature for 30 min. Unbound dye was removed using non-sterile tap water in a volume of 400 mL. After, the plates were dried until the water was removed entirely. The dye was extracted for 30 min, with 200 μL of 96% ethanol. The optical density of the CV solution was measured at 540 nm.

### 2.10. Statistical Data Analysis

Available experimental data were processed and evaluated mathematically and statistically directly in the Qinslab database. The exclusion of extreme values for data sets was performed by calculation in the Grubbs test. Experimental work was performed in at least three independent experiments (n_1_). Each sample in the experiments was analyzed at least five times (n_2_). The data are presented as average values. LOD (limit of detection) values were determined according to the work of Hubaux and Vos [71] at a significance level of 95%. Half-inhibition of growth (IC_50_) was determined by logit analysis using the HelpersMG (R) package. Cell growth intensity was calculated from the growth curve integrals. Lowering of growth intensity concentration compared to control was defined as inhibition described in percentages. The logit regression model was built using data obtained by growth curve analysis from all experiments. IC_50_ was calculated from the model as a theoretical value. Data visualization was performed using the Qinslab database. The number of asterisks (*) in graphs show the significance level in the following way: (*) *p* < 0.05, (**); *p* < 0.01 and (***); *p* < 0.001 and (x) *p* > 0.05.

## 3. Results

### 3.1. Characterization of Silver Nanoparticles (AgNPs)

**Figure 3:**
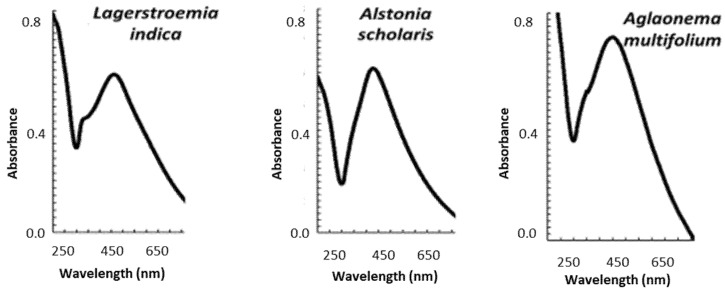
Characterization of AgNPs prepared using extracts from *L. indica* (LI), *A. scholaris* (AS), and *A. multifolium* (AM): Typical absorption spectra of each AgNPs. Other experimental details are in the section Material and Methods and Table 1.

Using green synthesis, AgNPs were synthesized from three plant extracts: *L. indica* (AgNPs_LI), *A. scholaris* (AgNPs_AS), and *A. multifolium* (AgNPs_AM) and characterized by transmission electron microscopy (TEM) and energy-dispersive X-ray spectroscopy (EDX) analysis (Table 1). Mostly spherical nanoparticles were obtained with sizes ranging from 10 to 40 nm, with an average diameter of 12, 15 nm, and 25 nm, respectively. Spherical AgNPs showed only one peak. An absorption maximum was detected at wavelength 450 nm for all nanoparticles (Figure 3). The more the absorption maximum was shifted to higher wavelengths, the larger the AgNPs were. The appearance of peaks with a smaller area under the curve at lower wavelengths confirmed the presence of monodisperse nanoparticles. On the other hand, peaks with a larger area under the curve at higher wavelengths confirmed the presence of polydispersion [72].

According to the results of the chemical analysis, AgNPs_LI had at least 70% silver; unlike other types of nanoparticles, they contained a relatively large amount of oxygen in the composition (Table 1). AgNPs_AS had the smallest amount of chlorine in the composition and contained at least 83% silver. AgNPs_AM had the highest carbon and chlorine content but the lowest silver concentration (at least 62%).

In further experiments, AgNPs were characterized in terms of secondary metabolites bound to their surface (Table 2). Silver nanoparticles were dispersed in ultrapure water, with 40 W ultrasound (ultrasonic cleaner USC-TH, VWR (Radnor, PA, USA), 30 °C) for 60 min, and then analyzed. Chemical analysis (total phenolic content, ABTS (2,2′-azino-bis(3-ethylbenzothiazoline-6-sulfonic acid)), DPPH(2,2-diphenyl-1-picrylhydrazyl) and FRAP (ferric reducing antioxidant power)) showed the presence of phenolic groups on the AgNPs surface. The total concentrations of phenolic compounds and the ability of AgNPs to quench free oxygen radicals are summarized in Table 2. Silver nanoparticles from *L. indica* extract (AgNPs_LI) had the least antioxidant activity in ABTS, FRAP, and DPPH tests. AgNPs_LI showed the largest number of phenolic compounds on the surface of nanoparticles; the lowest content of phenolic components was found in the AgNPs_AM sample. These data do not correlate with the amount of carbon in the nanoparticle composition; most likely, AgNPs_AM are modified by organic compounds without a phenolic ring, which also may have an influence on AgNPs antioxidant activity. Presumably, this role can be played by polysaccharides, the high content of which is noted for *A.* spp.; it is also noted that polysaccharides of plant extracts have significant antioxidant activity [73,74].

### 3.2. Antibacterial Effect of Silver Nanoparticles (AgNPs)

**Figure 4:**
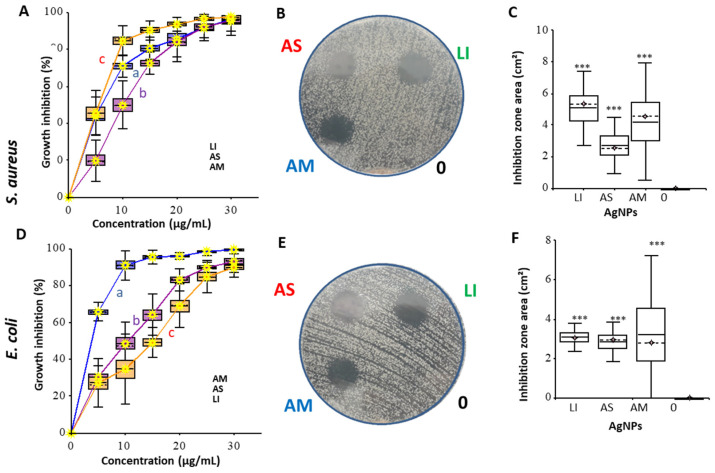
Effect of AgNPs prepared using extracts from *L. indica* (LI), *A. scholaris* (AS), and *A. multifolium* (AM) in liquid medium (**A**,**D**), in agar diffusion test for different (50, 25, 12.5, 6.25 µg/mL) concentrations (**B**,**E**) *S. aureus* and *E. coli* cells. Control in the diffusion test was marked as 0. (**C**,**F**) represent summary data from all performed integrals of inhibition surfaces. All measurements and experiments were performed in no less than five replicates. For further details, see the chapter Material and Methods.

The antibacterial effect of AgNPs was tested using a diffusion test on agar medium plates and in a liquid medium. The growth of bacterial culture in the presence of different concentrations of nanoparticles was detected by growth curves (OD_620_). *S. aureus* strain was used as a Gram-positive model; *E. coli* strain was chosen for an experimental Gram-negative model. The results are presented in Figure 4.

For MIC determination (Table 3), a dilution test was used; the logit model was performed using the HelpersMG package from growth curves integrals data. In the case of *S. aureus*, the best effect was shown for nanoparticles from *L. indica* (AgNPs_LI); a comparable effect, especially in agar diffuse tests, was also shown for AgNPs_AM. The best antibacterial effect on *E. coli* cells is shown for nanoparticles from *A. multifolium* (AgNPs_AM). AgNPs_LI and AgNPs_AS, according to the inhibition curve and diffusion test results on *E. coli* cells, have a similar effect on cells; however, the minimum inhibitory concentration (MIC) and theoretical values obtained from the models determine the lowest antibacterial effect of AgNPs_LI.

### 3.3. Antibiofilm Effect of Silver Nanoparticles (AgNPs)

**Figure 5:**
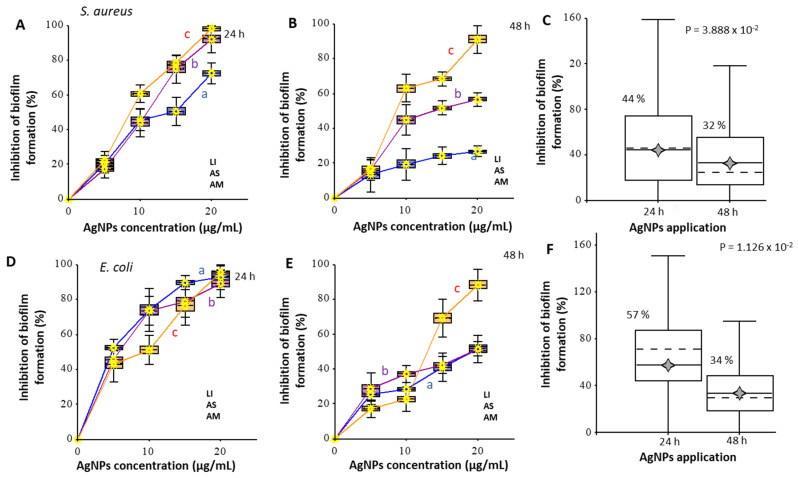
The antibiofilm potential of AgNPs prepared using extracts from *L. indica* (LI), *A. scholaris* (AS), and *A. multifolium* (AM) on *S. aureus* (**A**–**C**) and *E. coli* (**D**–**F**) cells after exposure 24 and 48 h. The effect was calculated relative to the control without nanoparticles (0%); calculations also considered the intrinsic density of nanoparticles adsorbed on the surface of the plate wells. A boxplot reflects the cumulative effect of all concentrations of one type of particle in an experiment. LB medium, incubation at 37 °C, staining with crystal violet. All measurements were performed in no less than five replicates. For further details, see the chapter Materials and Methods.

The effect of AgNPs on the formation of biofilms was studied. The density of biofilms was evaluated using crystal violet (CV) staining (Figure 5). In the case of *S. aureus* biofilms, the effect for different nanoparticles at a concentration of 5 μg/mL with an incubation time of 24 h did not have significant differences (*p* > 0.05), while the inhibition of biofilm growth was not more than 20%. The lowest effect was shown for AgNPs_AM; at the maximum concentration in the experiment (20 μg/mL), the effect did not exceed 70%, while AgNPs_LI and AgNPs_AS inhibited biofilm formation by more than 90%. An increase in cultivation time up to 48 h reduced the efficiency of AgNPs_AS and AgNPs_AM to inhibit the growth of biofilms by more than 50% at 20 μg/mL concentration; at 10 μg/mL concentration the effect did not significantly differ (*p* > 0.05) from the effect of maximum concentration. AgNPs_LI at the concentration of 20 μg/mL prevented the formation of *S. aureus* biofilm after 24 and 48 h of cultivation. All nanoparticles had a significant effect on the formation of *E. coli* biofilms after 24 h of biofilm growth in medium with AgNPs; at the highest concentration (20 μg/mL), the growth of biofilms decreased by more than 85%. The most significant effect at the concentration of 5 μg/mL was exerted by nanoparticles from *A. scholaris* (AgNPs_AS) and *A. multifolium* (AgNPs_AM); biofilm growth was inhibited by 50%. As in the case of *S. aureus* biofilm growth, after cultivation time increased from 24 h to 48 h, the effect of nanoparticles on *E. coli* biofilm growth was halved, except for nanoparticles from *L. indica* (AgNPs_LI) at the concentration of 20 μg/mL, the effect of which decreased by only 10%.

**Figure 6:**
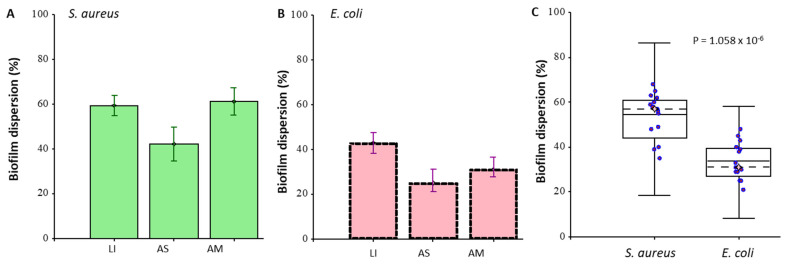
The ability of AgNPs prepared using extracts from *L. indica* (LI), *A. scholaris* (AS), and *A. multifolium* (AM) to disperse 48 h biofilms of *S. aureus* (**A**) and *E. coli* (**B**). Biofilm formation from all AgNPs analyzes in SA and EC (**C**). Absolute values for *S. aureus* OD_600_ = 0.40 ± 0.05 for *E. coli* OD_600_ = 0.70 ± 0.05. The effect was calculated relative to the negative control sample without nanoparticles (0%); calculations also considered the intrinsic density of nanoparticles adsorbed on the surface of the plate wells. LB medium, incubation at 37 °C. The staining was performed with crystal violet. All measurements were performed in no less than five replicates. For further details, see the section of Material and Methods.

The ability of AgNPs to destroy the formed biofilm of bacteria was investigated (Figure 6). Biofilms were grown for 48 h on polystyrene plates, and the medium was removed after incubation. Then, a solution of nanoparticles at a concentration of 20 μg/mL was placed in the well. After this, the biofilm with AgNPs was incubated for 24 h. The effect was calculated relative to the control sample without nanoparticles. The calculations also considered the intrinsic density of the nanoparticles adsorbed on the surface of the plate wells.

**Figure 7:**
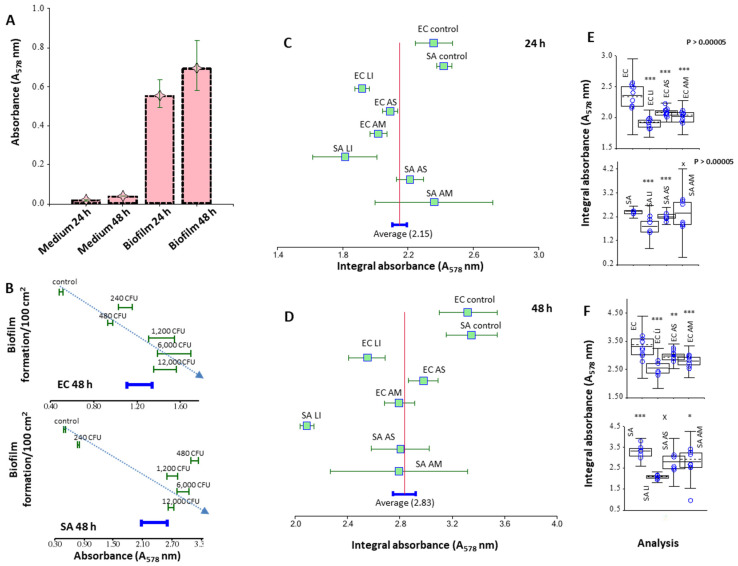
Spectrophotometric study of biofilm formation—Pyrogallol red method. Comparison of protein content in biofilm in 24 and 48 h against bacterial medium for *E. coli* (**A**). There are significant differences between protein content in media and biofilms and between 24 h and 48 h-biofilm. (**B**) Dependence of biofilm formation on CFU after 48 h. The protein content is increasing with a growing number of CFU. (**C**) Forest plot comparing the effect of using 20 μg/mL of AgNPs from *L. indica* (LI), *A. scholaris* (AS), and *A. multifolium* (AM) on biofilms of *S. aureus* (SA) and *E. coli* (EC) against non-treated biofilms in 24 h and (**D**) 48 h. (**E**) Boxplots comparing the overall area of non-treated biofilms and biofilms treated with 20 μg/mL AgNPs from LI, AS, and AM during 24 h and (**F**) 48 h.

AgNPs_LI destroyed *S. aureus* biofilms by 60% and the effect on *E. coli* biofilms was also destroyed by 42%. AgNPs_AS reduced the density of *S. aureus* and *E. coli* biofilms by 42 and 25%, AgNPs_AM by 63, and 33%, respectively. Thus, *S. aureus* biofilms were more susceptible to the treatment with AgNPs. However, we associate this with a total density of biofilms (when staining with CV, *E. coli* biofilms were 70% denser). The most destructive effect on biofilms was shown for AgNPs_LI. By measuring the total protein suspension of a dispersed biofilm, the effect of nanoparticles was studied. As a validation of the method, the dependence of the protein concentration in suspension on the starting cell concentration during biofilm growth and the background signal value (negative biofilm control) are shown. Using pyrogallol, reaction proteins were measured in a suspension of a dispersed biofilm, which was grown in the presence of silver nanoparticles at a concentration of 20 μg/mL (Figure 7). Biofilms were grown in polystyrene Petri dishes, so the adhesion area was significantly increased. The greatest effect was shown for AgNPs_LI, which amounted to 23 and 19% (for 24 h growth), 37 and 25% (for 48 h growth) for *S. aureus* and *E. coli* biofilms, respectively, compared to the control (nontreated biofilm). On average, an incubation effect of 48 h caused less biofilm growth, relative to incubation of 24 h, which also corresponds to the results of CV staining. The effect of AgNPs_AS and AgNPs_AM on bacterial biofilms was 10–15%.

**Figure 8:**
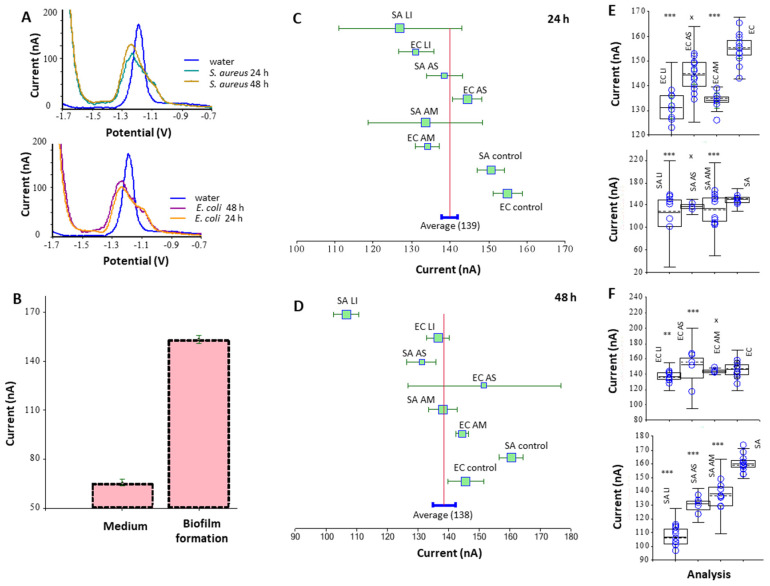
Electrochemical study of biofilm formation—the Brdicka reaction. A range of measurement: −0.7 V to −1.95 V; voltage amplitude: −25 mV; time amplitude: 0.8 s; measurement time: 10 ms; pulse time: 30 ms; voltage step: 4 mV; sweep rate: 5 mV/s; sample volume: 10 μL; Brdicka buffer volume: 3 mL. Dependence of electrical current (nA) on potential (V). Mutual curve comparison between water and biofilm formation in the first and the second day for *E. coli* (EC) and *S. aureus* (SA) (**A**). Comparison between absolute area signals among medium and biofilm (**B**). Forest plot comparing effect of using 20 μg/mL of AgNPs from *L. indica* (LI), *A. scholaris* (AS), and *A. multifolium* (AM) on biofilms of *S. aureus* and *E. coli* against non-treated biofilms in 24 h (**C**) and 48 h (**D**). Box-plots comparing the overall area of non-treated biofilms and biofilms treated with 20 μg/mL AgNPs from LI, AS, and AM during 24 h (**E**) and 48 h (**F**).

Additionally, the measurement of protein in a suspension of a dispersed biofilm was studied using the Brdicka electrochemical method (Figure 8). The method was also validated by measuring biofilm growth at 24 and 48 h. The effect of AgNPs at a concentration of 20 mg/mL was studied in relation to the control (untreated biofilm); the greatest effect was shown for AgNPs_LI nanoparticles and the effect for *S. aureus* and *E. coli* was 16 and 13% (for 24 h incubation), 33 and 6% (for 48 h incubation), respectively. AgNPs_AM had no significant effect on *E. coli* biofilms (*p* < 0.05) for 48 h; in general, the effect of the tested AgNPs on *E. coli* biofilms was less than on *S. aureus* biofilms.

## 4. Discussion

This study demonstrated the antibacterial activity of green-synthesized AgNPs against both Gram-positive and Gram-negative strains. The antibacterial effect of AgNPs investigated using a diffusion test and growth curves (Figure 4, Table 3) was found in AgNPs_LI and AgNPs_AM against *S. aureus* and *E. coli*, respectively. Gupta et al. [75] phyto-synthesized AgNPs that showed broad-spectrum anti-microbial activity against Gram-positive, Gram-negative pathogenic bacteria, and fungi such as *Candida albicans*. In the study by Bharathi et al. [76] based on the agar well diffusion method, AgNPs exhibited significant antibacterial activity against *S. aureus* and *E. coli*. In the study by Singh et al. [77], AgNPs biosynthesized using *Penicillium* sp. isolated from healthy leaves of *Curcuma longa* exhibited antibacterial activity against multidrug-resistant pathogens *E. coli* and *S. aureus,* based on the diffusion assay method. The maximum zone of inhibition was of 17 mm and 16 mm, respectively, at 80 µL of AgNPs used. Similar effects on *E. coli* and *S. aureus* were reported by Kim et al. [38] in AgNPs prepared by using the chemical method, as well as by Ninganagouda et al. [78], who used AgNPs synthesized by *Aspergillus flavus*.

Minimum inhibitory concentrations (MICs) of tested AgNPs (Table 3) ranged between 15 and 20 μg/mL for both monitored bacteria. In a study by Qayyum et al. [79], AgNPs synthesized using Mangifera indica inflorescence aqueous extract exhibited MICs of 8 μg/mL and 16 μg/mL for *E*. *coli* and *S*. *aureus* strains, respectively, which was relatively quite low compared to chemically synthesized AgNPs.

The AgNPs prepared by utilizing an aqueous extract of *Cannabis sativa* stem inhibited *E. coli* biofilm. They showed MIC value of 5 µg/mL and minimum bactericidal concentration value of 25 µg/mL against this bacterium [19].

The effect of AgNPs on the inhibition of biofilm formation was determined using the density of biofilms evaluated using crystal violet assay (Figure 5). The *S. aureus* biofilm formation was inhibited by more than 90% when AgNPs_LI or AgNPs_AS at the concentration 20 μg/mL were added. In the case of *E. coli*, all tested AgNPs had a significant inhibition effect on the formation of biofilm after 24 h; the AgNPs concentration of 20 μg/mL reduced the biofilm growth by more than 85%. In the study by Singh et al., AgNPs inhibited 80% biofilm of *E. coli*, based on quantitative observation by crystal violet assay [19]. In the study by Gupta et al. [75], phyto-synthesized AgNPs exhibited extraordinary ability to inhibit the biofilms formed by *S. aureus* and *E. coli* determined by crystal violet assay. Bharathi and Bhuvaneshwari [76] observed the antibacterial and anti-biofilm activity of AgNPs synthesized using *Cordia dichotoma* fruits. Phyto-synthesized AgNPs showed more than 90% inhibition of biofilm activity formed by *S. aureus* and *E. coli*.

Barabadi et al. [80] evaluated the antibacterial activity and biofilm inhibitory activity of AgNPs prepared by biosynthesis using the aqueous extract of *Zataria multiflora* compared to commercial AgNPs. The plant mediated fabricated AgNPs and commercial AgNPs exhibited significant antibacterial activity with MIC of 4 and 8 µg/mL against *S. aureus*, respectively. Both types of AgNPs at the concentrations of 4*MIC resulted in the complete avoidance of biofilm formation. Significant biofilm inhibitory activity was observed at the concentrations of ≥8 µg/mL in both types of AgNPs. However, at the lower concentrations, the green-synthesized AgNPs exhibited greater biofilm inhibition formed by *S. aureus*.

*Staphylococcus aureus* is considered by the World Health Organization to be a high priority pathogen for which new therapeutic options need to be developed. This is especially important for biofilm implant-associated infections if the only treatment option available is surgery combined with antibiotic therapy [81]. Silver nanoparticles (as a composite with graphene oxide) could be a promising material to control nosocomial infections caused by bacteria strains, which are resistant to antibiotics [82]. mPEGTH2-AgNPs have been demonstrated as a promising candidate to kill pathogenic microbes [68].

Nanoparticles are possible to have a more significant effect on Gram-negative cells [35]. This can explain the higher inhibitory effect of AgNPs in low concentrations on the growth of *E. coli* biofilms, compared with *S. aureus* biofilm. In the case of the destruction of the biofilm, it seems that the results of the experiment can be described by differences in biofilm formation properties of strains: density of mature biofilms, rate of division and formation of microcolonies, etc.

Salunke et al. [83] reported a connection between nanoparticles and their several physicochemical properties in action on different bacterial strain’s biofilms. Additionally, in our study, the effect of the green synthesized AgNPs on bacterial biofilms was demonstrated. This effect correlated, to a greater extent, with the results of the diffusion test (Figure 4) than with the MIC (Table 3). AgNPs had a generally lower effect on *E. coli* compared to *S. aureus* biofilm (Figure 8).

The inhibitory concentration is a characteristic that primarily indicates the susceptibility of bacteria to a specific type, modification or size of nanoparticles [83]. Some researchers emphasize that smaller nanoparticles can have a more significant effect on biofilms because they quickly diffuse into the three-dimensional structure of biofilms and can affect a more significant number of cells [84]. The size of all studied nanoparticles in our work was comparable. However, as demonstrated by the TEM results, AgNPs_AS particles formed denser aggregates in the solution. This explains the low efficiency of these nanoparticles in the destruction of mature biofilms (Figure 6) and lower diffusion in the antibacterial test on an agar medium.

Earlier, the toxic effect of zinc nanoparticles synthesized from extracts of *A. scholaris* was reported. For the most part, the prevalence of the antifungal effect was shown, compared with the antibacterial effect [85]. Thanks to the combined effect of the components of *A. scholaris* extract and the antibacterial and anti-inflammatory effects, the nanoparticles that can be synthesized from this plant can be optimized for use in the treatment of wounds. In the case of AgNPs, the toxic effect of AgNP on eukaryotic cells is also considered [50,53,56]. A promising critical approach to improving the antibiofilm and antimicrobial properties of nanoparticles is the use of individual components of plant extracts. For example, 3,5,7-trihydroxyflavone present in *A. scholaris* extract disrupt the functioning of quorum sensing in Gram-negative bacteria [86]. Diab et al. [52] reported a compound from the methanol extract of *L. indica* which was characterized as ′4-methoxy apigenin-8-C-β-D-glucopyranoside with a high antimicrobial effect. Moreover, a promising approach with individual components could be simulated first-hand with the molecular docking protocol to observe the binding between the AgNPs and the protein with in silico visualization.

In the article by Kriswandini et al., the measurement of the total biomass of proteins in biofilms (cells and matrix) was analyzed not only quantitatively, but also qualitatively by electrophoresis [87]. Additionally, the total biofilm protein is measured in conjunction with dry biomass biofilm as well as to normalize data from other biofilm tests [88]. Measurement of the protein in the composition of the destroyed biofilm allows you to obtain information about cells and extracellular proteins can be used as an indicator of the effect of antimicrobial substances on the state of the system as a whole, including used in conjunction with CFU.

Using the electrochemical Brdicka method (Figure 8), which was mainly used previously to measure thiols and small stress proteins [61,89], as another method of measuring protein in a biofilm, correlates well with the results of the pyrogallol reaction (Figure 7). In general, the biofilm measurement methods used in this work are relatively related.

Summarizing our observation, as well as the results of previous studies, various authors have shown that AgNPs can be considered as powerful antibacterial agents against *E. coli* and *S. aureus.* The most important mechanism of bacterial inhibition by AgNPs can be attributed to ROS. ROS may be one of the possible modes of action of AgNPs. AgNPs generated ROS when interacting with bacterial cells. AgNPs interaction with bacterial membrane led to its damage [79].

## 5. Conclusions

Tropical forests have great potential in the search for new biologically active substances of plant origin that could be used to treat serious human diseases. In this study, AgNPs were prepared by the reduction in plant extracts (*Lagerstroemia indica*, *Alstonia scholaris*, *Aglaonema multifolium*) with total phenol content (270–380 mg/mL). The formed nanoparticles showed antibacterial activity against model microorganisms *S. aureus* and *E. coli*. The MICs was found ranged from 15 to 20 μg/mL. In addition, these nanoparticles have been applied to bacterial films formed by the above-mentioned bacteria. We found that the nanoparticles were able to disrupt these films effectively after 24 h (44% and 57% for *S. aureus* and *E. coli*, respectively) and after 48 h (32% and 34% for *S. aureus* and *E. coli*, respectively); however, the effect was reduced by about half. In addition, levels of thiol compounds, especially metallothionein, were monitored (increase in total metallothionein (MT) levels from 70 to 150 nA). The MT of the microorganism can both modify the nanoparticles and bind silver ions, thus significantly reducing the biological activity of these particles. Therefore, it is necessary to address this issue and recognize other pathways of bacterial resistance to heavy metals/metal ions as antibacterial agents further intensively.

## Figures and Tables

**Table 1:** Energy-dispersive X-ray spectroscopy (EDX) analysis of AgNPs from *L. indica* (LI), *A. scholaris* (AS), *A. multifolium* (AM). All measurements were performed in no less than five replicates.

wt%	AgNPs_LI	AgNPs_AS	AgNPs_AM
**C**	8.95 ± 0.90	9.78 ± 0.25	22.90 ± 0.37
**O**	17.22 ± 1.16	5.35 ± 0.33	6.09 ± 0.30
**Cl**	2.79 ± 0.18	0.92 ± 0.05	8.31 ± 0.08
**Ag**	70.13 ± 1.24	83.95 ± 0.38	62.70 ± 0.37

**Table 2:** Biochemical characterization of AgNPs prepared using extracts from *L. indica* (AgNPs_LI), *A. scholaris* (AgNPs_AS), and *A. multifolium* (AgNPs_AM). (a) Concentrations of total phenolic content on the surface of AgNPs. The ability of AgNPs to quench free oxygen radicals—(b) ABTS, (c) DPPH, and (d) FRAP methods. GAE—gallic acid equivalent. All measurements were performed in no less than five replicates.

	Total Phenol Content	DPPH	ABTS	FRAP
AgNPs	(mg/mL, GAE)			
LI	380 ± 20	0.12 ± 0.05	0.15 ± 0.10	1.40 ± 0.15
AS	300 ± 15	0.15 ± 0.05	0.90 ± 0.15	2.00 ± 0.20
AM	270 ± 10	0.32 ± 0.10	0.4 ± 0.10	1.95 ± 0.15

**Table 3:** Minimal inhibitory concentrations (MICs), Logit LC_50_ (lethal concentration) model values, and LOD (limit of detection model) from a diffusion test as antimicrobial characteristics of AgNPs prepared using extracts from *L. indica* (AgNPs_LI), *A. scholaris* (AgNPs_AS), and *A. multifolium* (AgNPs_AM).

Strain	*S. aureus*			*E. coli*		
Nanoparticles	AgNPs_LI	AgNPs_AS	AgNPs_AM	AgNPs_LI	AgNPs_AS	AgNPs_AM
MIC, µg/mL	15.0 ± 5.0	20.0 ± 5.0	20.0 ± 5.0	20.0 ± 5.0	15.0 ± 5.0	15.0 ± 5.0
Logit LC_50_, µg/mL	7.0 ± 0.4	12.0 ± 0.6	12.0 ± 0.6	15.0 ± 0.8	9.0 ± 0.5	5.0 ± 0.3
LOD, µg	25.0 ± 1.3	37.0 ± 2.0	19.0 ± 1.0	34.0 ± 2.0	30.0 ± 2.0	16.0 ± 1.5

## Data Availability

Data is contained within the article.
